# Characterization of Fast-Scan Cyclic Voltammetric Electrodes Using Paraffin as an Effective Sealant with *In Vitro* and *In Vivo* Applications

**DOI:** 10.1371/journal.pone.0141340

**Published:** 2015-10-27

**Authors:** Eric S. Ramsson, Daniel Cholger, Albert Dionise, Nicholas Poirier, Avery Andrus, Randi Curtiss

**Affiliations:** Biomedical Sciences Department, Grand Valley State University, Allendale, MI 49401, United States of America; University of Leicester, UNITED KINGDOM

## Abstract

Fast-scan cyclic voltammetry (FSCV) is a powerful technique for measuring sub-second changes in neurotransmitter levels. A great time-limiting factor in the use of FSCV is the production of high-quality recording electrodes; common recording electrodes consist of cylindrical carbon fiber encased in borosilicate glass. When the borosilicate is heated and pulled, the molten glass ideally forms a tight seal around the carbon fiber cylinder. It is often difficult, however, to guarantee a perfect seal between the glass and carbon. Indeed, much of the time spent creating electrodes is in an effort to find a good seal. Even though epoxy resins can be useful in this regard, they are irreversible (seals are permanent), wasteful (epoxy cannot be reused once hardener is added), hazardous (hardeners are often caustic), and require curing. Herein we characterize paraffin as an electrode sealant for FSCV microelectrodes. Paraffin boasts the advantages of near-immediate curing times, simplicity in use, long shelf-life and stable waterproof seals capable of withstanding extended cycling. Borosilicate electrode tips were left intact or broken and dipped in paraffin embedding wax. Excess wax was removed from the carbon surface with xyelenes or by repeated cycling at an extended waveform (-0.4 to 1.4V, 400 V/s, 60 Hz). Then, the waveform was switched to a standard waveform (-0.4 to 1.3V, 400 V/s, 10 Hz) and cycled until stable. Wax-sealing does not inhibit electrode sensitivity, as electrodes detected linear changes in dopamine before and after wax (then xylenes) exposure. Paraffin seals are intact after 11 days of implantation in the mouse, and still capable of measuring transient changes in *in vivo* dopamine. From this it is clear that paraffin wax is an effective sealant for FSCV electrodes that provides a convenient substitute to epoxy sealants.

## Introduction

Fast-scan cyclic voltammetry (FSCV) allows for the real-time measurement of various neurotransmitters, including dopamine (DA), serotonin, norepinephrine, and histamine during behavior [1–4]. In order to measure these substances, small cylinders of carbon fiber (typically 5–7 *μ*m in diameter; 10–200 *μ*m in length) are utilized to force redox reactions and measure the resulting electron gain or loss. These redox reactions result from charging the carbon surface with various ramp voltage signals [4–7]. In order for proper surface charging and to limit reaction location, it is critical that only a small portion of carbon cylinder is exposed to the recording environment, while any remaining carbon is properly insulated from reaction. Typically, the remaining carbon extends either within borosilicate glass capillary tubing (from herein termed simply “glass”), or through fused-silica capillary tubing. In the case of glass electrodes, within the tubing itself electrical connection is made between the carbon fiber and stainless steel wiring using metal alloys such as bismuth [8–10], high salt solutions such as KCl [11], colloidal graphite [12], or a resin-carbon powder mixture [13]. Fused-silica electrodes (FSE), which are used for chronic implantation, utilize an electrical contact of silver epoxy or silver paint [14]. The internal and external environments must be properly insulated from one another, or capillary action will draw external solution and cause spurious reactions and extensive electrode drift/instability.

Traditionally, the separation of internal and external carbon surfaces has relied upon either molten glass sealing around the carbon surface [8, 9], or epoxy resins forming the glass/fused-silica/carbon fiber seal [1, 12, 14–16]. At times these methods are combined, and the epoxy resin is used to “fill in the gaps” of the glass/carbon seal [17]. This is because the glass/carbon sealing relies upon the proper heating and pulling of a micropipette puller [18], which can be sensitive to humidity, filament age, small changes in filament orientation, and changes in pull strength exerted upon the molten glass. Additionally, use of FSEs requires the use of epoxy sealants to close the open tubing around the carbon fiber sensor [14].

While epoxy has long been used as a glass/carbon sealant [13], epoxy resins have several drawbacks that warrant the seeking of additional sealants. Epoxy resins often have limited working times, and once epoxy has hardened, it cannot be reused. This also means than once an epoxy seal has occurred between glass and carbon, this seal cannot be changed should it prove inadequate. In addition, epoxy resin hardeners are frequently caustic. Lastly, should epoxy resin not adequately be removed from the carbon surface before hardening, the reactive surface is now insulated and the electrode is inert.

Herein, we demonstrate paraffin wax as an adequate insulator for the creation of FSCV cylinder electrodes. It boasts unlimited working times (as long as a heat source is maintained), can be reheated and reused almost indefinitely, and an improper seal can be changed by reheating and re-dipping the electrode. Should the carbon surface be insulated by too much wax, additional dipping removes much of this surface wax; any remaining wax can be removed with xylenes or cycled off the surface [5]. Paraffin wax/carbon seals withstand repeated cycling at the electrode surface; the seal remains intact after the equivalent of 24 hours of regular cycling (-0.4 to 1.3 V and back, 400 V/s, 10 Hz). Importantly, wax seals are stable at normal mammalian body temperature, allowing for detection of DA in a freely-moving mouse up to 11 days after implantation.

## Materials and Methods

### Electrode Construction

Fast-scan cyclic voltammetry (FSCV) carbon fiber glass microelectrodes were created by using vacuum to draw carbon fiber (7 *μ*m in diameter, T-650, Cytec Engineering, Woodland Park, NJ) into glass capillary tubes (1.2 mm O.D.; A-M systems, Sequim, WA) which were then pulled in a horizontal electrode puller (P-77 Sutter Flaming-Brown Micropipette Puller, Sutter Instruments, Novato, CA). Electrode tips were left intact (glass-sealed) or broken under a stereo microscope to ensure a small-diameter break perpendicular to the longitudinal axis. Embedding paraffin (Sigma-Aldrich, St. Louis, MO) was melted in a crucible with the heat gun of a Kendal Rework Soldering Station (via Amazon.com) or regular heat gun (Master Appliance Corp., Racine, WI). Electrodes were then dipped in the molten wax. Electrodes dipped in xylenes (Sigma-Aldrich, St. Louis, MO) were swirled for ∼1–3 s. Carbon fiber was trimmed by scalpel under a stereomicroscope to 150–250 *μ*M in length. Electrical connection was made between the carbon fiber and a stainless-steel wire by 1 M KCl solution.

*In vivo* fused-silica electrodes (FSE) were fabricated by threading (in ethanol) carbon fiber through polyimide-covered fused-silica tubing (Polymicro Technologies, Phoenix, AZ) and allowing them to dry overnight. The next day, both the carbon fiber and fused-silica were dipped in molten wax. Electrical connections were made by connecting the remaining carbon fiber (on the side opposite to the wax seal) to a silver pin (Newark, Palatine, IL) using silver epoxy.

### Chemicals

All chemicals were purchased from Sigma Aldrich (St. Louis, MO). Stock solutions of dopamine HCl (10 mM) were made in 0.1 N perchloric acid and diluted to the desired concentration the day of experiment in artificial cerebrospinal fluid (aCSF) described previously [19] and comprised of (in mM): 125 NaCl, 4 KCl, 1.3 CaCl_2_, 1 MgCl_2_, 0.66 NaH_2_PO_4_, 2 Na_2_HPO_4_, 1 glucose, pH 7.4. All solutions were made in 18 mOhm water (Barnstead E-Pure, Thermo Scientific, Waltham, MA).

### Optical Microscopy

Microscopic images were taken with a Nikon Eclipse Ni upright microscope and Nikon DS-Fi2 camera (Tokyo, Japan).

### Electrochemical Instrumentation

Data was collected using a ChemClamp potentiostat (Dagan, Minneapolis, MN, with custom-modified gain settings [20]) controlled by the Demon Voltammetry and Analysis [20] program with the exception of *in vivo* recordings (see below). After electrodes were sealed with wax, they were cycled from -0.4 to 1.3 or 1.4 V and back (versus an Ag/AgCl reference) at a ramp of 400 V/s and application frequency of 60 Hz until stable (typically 15 min). Excursions at this frequency to 1.3 V have been shown to renew the carbon surface and reduce surface fouling [5] that may occur due to leftover thin layers of wax. If xylenes were not used to clean the carbon surface, then excursions to 1.4 were chosen to speed up this process, as excursions to 1.4 V renew the carbon surface faster [5]. Then, once stable (less than 2 nA drift over 30 s at 60 Hz), electrodes were cycled from -0.4 to 1.3 V and back at 400 V/s at a frequency of 10 Hz until stable (less than 2 nA drift per 30 s) prior to experiment.

### Extended Cycling

Paraffin-sealed electrodes were cycled for 4 hours at -0.4 to 1.3 to -0.4 (400 V/s; 60 Hz) in aCSF to evaluate seal stability.

### Flow Injection Analysis

Flow injection analysis was performed in a gravity-fed microfluidics system (hereafter “calibration system”) created in-house based upon the work done by Sinkala and colleagues [19]. aCSF alone or aCSF plus DA was sent by the electrode, and the latter occurred for approximately 15 seconds at a flow rate ∼3 mL/min. Flow of aCSF was created using gravity through two open 60 mL syringes, and flow rate was adjusted with an intravenous (IV) flow regulator (Wolf Medical Supply, Sunrise, FL). The flow from either syringe was stopped by an attached stopcock.

### Wax Melting Point

Melting point of the paraffin wax was determined via an MPA160 Melting Point Apparatus (Standford Research Systems, Sunnyvale, CA) to be 50–53°C.

### Animal Experiments

The use of animals was conducted within guidelines described by the Guide for the Care and Use of Laboratory Animals from the National Institutes of Health. All protocols involving animal use were approved by the Committee on the Ethics of Animal Experiments of the Salk Institute for Biological Studies. Animals were housed in a humidity- and temperature-controlled vivarium with a 12 hour light-dark cycle and food was provided ad libtum.

### Animals

One female mouse with a predominant C57BL/6J background (∼ 21g at the time of electrode implantation) was used to demonstrate the biocompatibility of wax sealants *in vivo*. The mouse was deeply anesthetized with a cocktail of ketamine and xylazine (100 mg/kg ketamine and 5 mg/kg xylazine injected intraperitoneally) and placed in a stereotactic apparatus (David Kopf Instruments, Tajunga, CA). A hole was drilled above the dorsal striatum (+0.8 AP, +1.5 ML [21]) and a wax-sealed fused-silica recording electrode was lowered to a final depth of -2.5 DV. A second hole was drilled contralaterally for implantation of a Ag/AgCl reference wire. Two skull screws were secured in the skull, and the electrode, the reference wire, and a bipolar electrode (Plastics One, Roanoke, VA) modified to connect to both the recording and reference electrodes, were fixed in place with dental cement. Following surgery, animal health was closely monitored and access to ibuprofen water (0.1 mg/ml) was provided.

### *In vivo* FSCV Recordings

Following implantation, the electrode and reference were connected to a headstage and potentiostat which were fabricated by the Department of Psychiatry at the University of Washington. FSCV recordings were conducted by applying voltage at the carbon fiber sensor from -0.4 V to 1.3 V relative to the Ag/AgCl reference and back at a rate of 400 V/s every 100 ms (10 Hz recording). Daily recordings were made using software written in LabVIEW (National Instruments, TX) to monitor electrode status. On the eleventh day, the mouse was placed in a Med Associates chamber [22] (Med Associates, St. Albans, VT) and a longer recording session was conducted where spontaneous changes in DA were assessed while the mouse freely explored the chamber.

### Data Analysis

Data analysis was done in the Demon Voltammetry software, and data manipulations were performed in Microsoft Excel. Data were graphed in Veusz (http://home.gna.org/veusz/) and compiled in the GNU Image Manipulation Program (GIMP; www.gimp.org). Brightfield microscopy images were enhanced for better contrast, rotated, and cropped. Statistical analyses were done in PAST (http://folk.uio.no/ohammer/past/). Statistical significance was set at *p* < 0.05.

## Results

### Fabrication of Wax-sealed Electrodes

**Fig 1:**
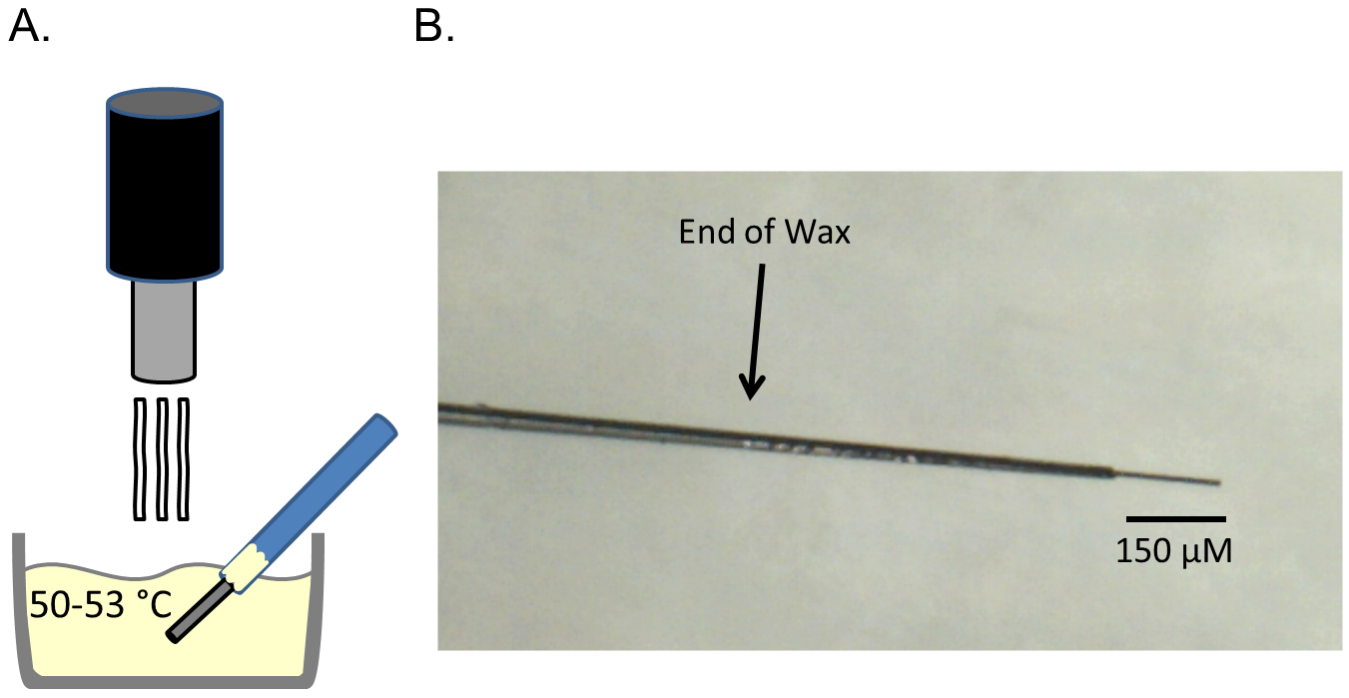
Creation of wax-sealed glass electrodes. Glass electrode tips were broken and then resealed in molten embedding paraffin, which wicks up the carbon fiber. A) Cartoon of this process, showing a heat gun used to melt embedding paraffin to between 50–53°Celsius, in which the broken-tipped electrode is dipped. B) Optical microscopy of a paraffin-sealed glass electrode. The carbon fiber can be seen to extend beyond the paraffin seal, and the seal can be seen to extend within the glass.

Fig 1A shows a cartoon representation of wax-sealed electrode creation using a top-down heat source and crucible full of wax. Once the glass seal is broken, the electrode is dipped in the molten wax. The carbon fiber allows the wax to quickly wick up into the capillary tubing. This wicking action can be seen in Fig 1B, where the wax has wicked up ∼ 6 mm within the tubing. A clearly defined seal end can be seen, and no visible wax droplets are apparent on the carbon fiber.

**Fig 2:**
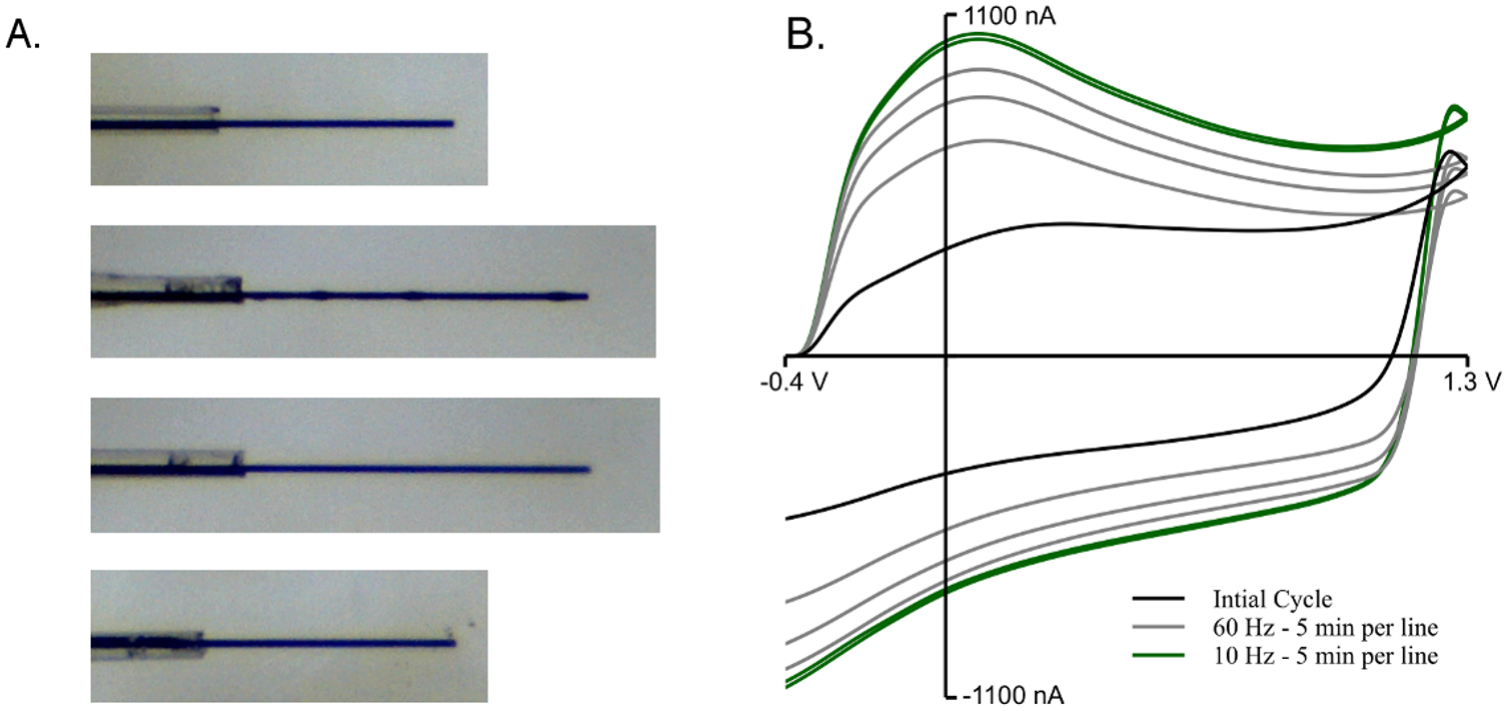
Detailed creation of wax-sealed glass electrodes. A Wax-sealed electrode was created in < 20 minutes (including photographs; not including cycling). A) After the electrode was pulled in a micropipette puller, the glass/carbon seal was broken using an empty capillary tube to crush the seal. This was repeated until a break perpendicular to the carbon fiber was achieved. The electrode was then dipped in molten wax, which drew the carbon fiber out an additional 103 *μ*m. Also, wax droplets can be seen on the carbon fiber surface. After photographing, the electrode was swirled in xyelenes for ∼ 1 s, which removed the visible wax droplets from the surface. Lastly, the electrode was cut using a scalpel to 245 *μ*m. The events described are depicted from top to bottom (carbon fiber lengths: 224, 327, 327, and 245 *μ*m from top to bottom, respectively). B) The same electrode in A was cycled in aCSF from -0.4 to 1.3 V and back at 400 V/s and 60 Hz application frequency for 15 minutes. Then, it was cycled at 10 Hz for 10 minutes. Data was collected for 30 s initially (black line), and then 30 s every 5 minutes thereafter. Gray lines indicate data collected after 5, 10 or 15 minutes at 60 Hz, while the green lines represent data collected after 5 or 10 minutes of 10 Hz application.

Fig 2 shows the creation steps in greater detail. After the carbon fiber-containing capillary was pulled, the glass seal was broken by crushing with another empty capillary tube while visualizing under a stereomicroscope (Fig 2A, top; 224 *μ*m exposed carbon). The electrode was dipped in wax (Fig 2A, 2nd from top), and it is apparent that as the wax wicked up the carbon fiber, the carbon fiber was drawn out slightly (new length 327 *μ*m), which is not uncommon. Wax droplets can be seen on the carbon fiber. Next, the electrode was swirled in xylenes for ∼ 1 s, which removed the wax droplets off of the carbon surface (Fig 2A, 3rd from top). Lastly, the carbon fiber was trimmed with a scalpel to 245 *μ*m (Fig 2A, bottom). All steps (including photographs) took < 20 minutes. It is important to note that the end of the wax line (as show in Fig 1B) cannot be seen in these images.

To determine seal stability, the electrode was cycled (in aCSF) as follows: -0.4 to 1.3 V and back, 400 V/s, 60 Hz for 15 minutes, collecting 30 s of data every 5 minutes (Fig 2B; gray lines). Then, the electrode was cycled at 10 Hz for 10 minutes, collecting 30 s of data every 5 minutes (Fig 2B; green lines). During the last data collection, electrode drift was ∼ 1.5 nA (data not shown). The combination of little increase in background charging current (BC) with small electrode drift is what we shall hereafter refer to as “stable” for electrodes. Although these cycling parameters work for many electrodes, each electrode is unique, and some of above steps (including dipping in wax and/or xylenes) may need repeating.

### Removal of Wax and Seal Stability

**Fig 3:**
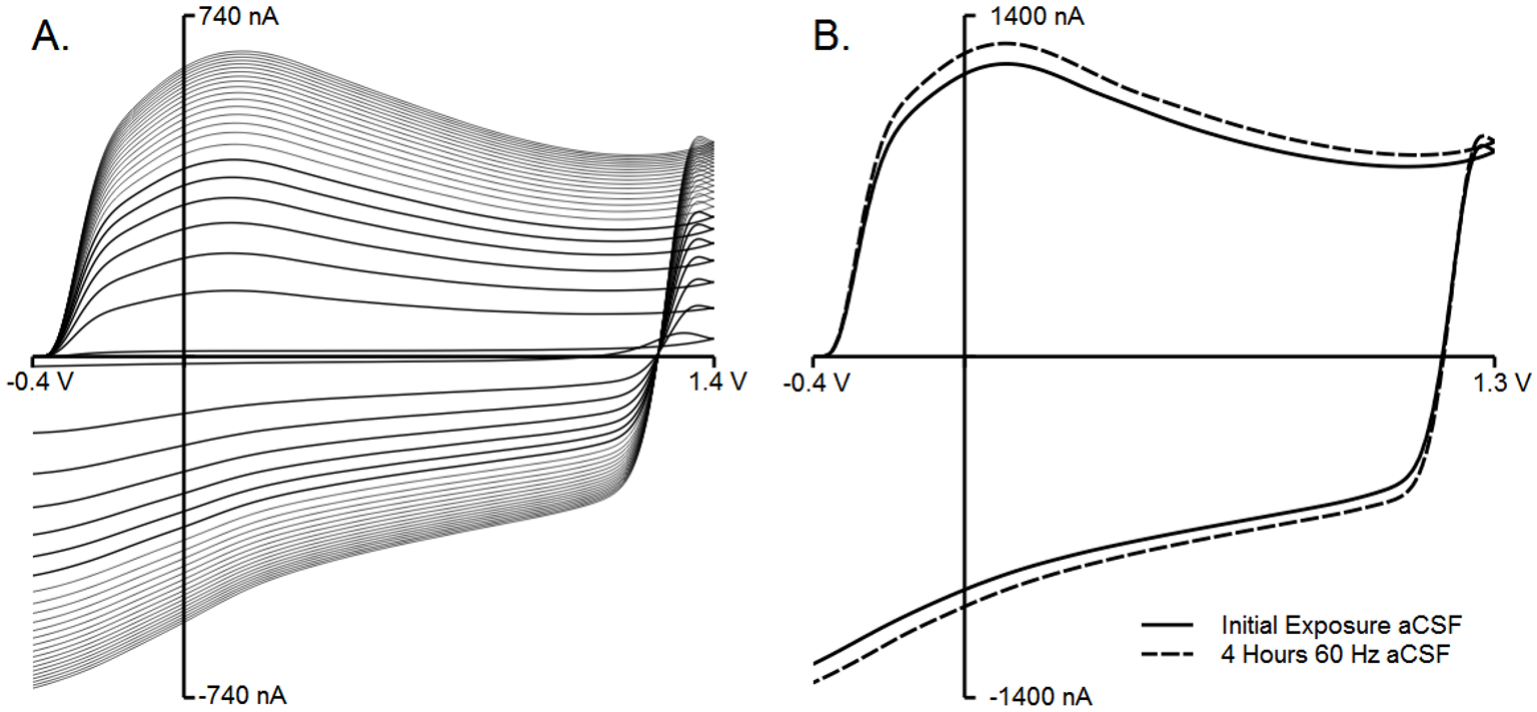
Removal of surface wax and seal stability. Any wax fouling the reactive carbon surface can be removed by electrochemical etching. A) A glass electrode sealed with embedding paraffin, with the carbon surface intentionally left insulated by wax. Cycling the electrode from -0.4 to 1.4 V and back (400 V/s; 60 Hz) removes surface wax, as indicated by an increase in BC of the electrode. The electrode was constantly cycling, and recordings occurred every five minutes, shown by each successive line. After 2 hours, the growth of the background current stopped, which is the largest background current recording. It can be seen that the growth rate successively slowed until stability is reached. B) A paraffin wax-sealed electrode was cycled at 60 Hz (triangle application every 16 ms) from -0.4 to 1.3 V and back at 400 V/s. Once the electrode stabilized, recordings were taken initially and after 4 hours. There is very little difference between the initial cycling and 4 hours later, which equates to 24 hours of continuous cycling at 10 Hz (every 100 ms). This shows that the wax seal is stable for extended cycling periods.

An electrode was intentionally left fouled with wax by not dipping it in xyelenes or re-dipping it in molten wax. Upon exposure to aCSF, the BC was greatly reduced (see line closest to x-axis). Cycling from -0.4 to 1.4 and back at 400 V/s (60 Hz) appears to etch the carbon surface and remove surface wax, as repeated cycling with this waveform caused the BC to steadily increase. Each successive increase in BC shown in Fig 3A (from smaller to larger) was recorded at 5 minute intervals for 2 hours. The longer the electrode cycled, the smaller the increase interval in BC (seen by smaller gaps between each line), indicating that the electrode eventually reached a stable state. To demonstrate continuing seal stability, more experimentally relevant waveform parameters [9, 10, 14, 23] were utilized (-0.4 to 1.3 V and back, 400 V/s) at a faster application (60 Hz) to facilitate data collection. Four hours of cycling (which equates to 24 hours of 10 Hz cycling) shows a slight increase in, but no loss of, BC (Fig 3B.

### Sensitivity to Dopamine Not Fouled by Wax

**Fig 4:**
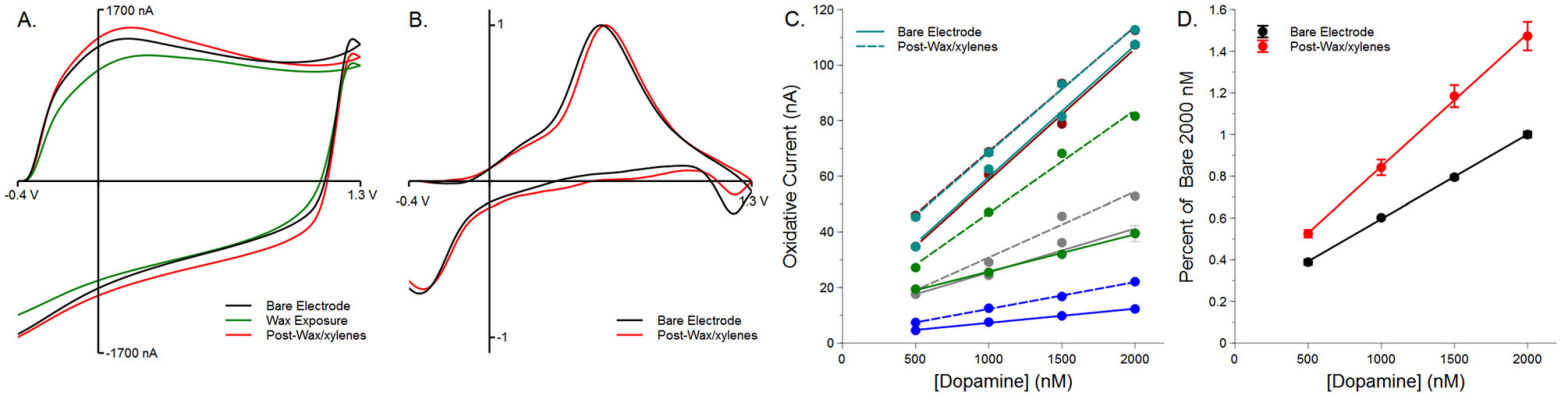
Exposure to wax does not apparently foul the reactive surface. Electrodes exposed to molten paraffin wax, then xylenes show an enhanced sensitivity to DA. A) Glass-sealed electrodes (in black: Bare Electrode) were exposed to molten paraffin and then swirled in xylenes for 1–3 seconds. Exposure to molten wax reduced the reactive surface of the electrode, presumably by depositing a thin layer of wax. This can be seen by a reduction in the background charging current (in green). However, exposure to xylenes restored the electrode to a larger-sized background current (in red: Post-Wax/xylenes). B) The oxidation peak for 1000 nM DA (normalized to own peak for comparative purposes) shows a minor (0.024 V) shift right after exposure to wax and then xyelenes (bare oxidation = 0.554 V; post-wax/xylenes = 0.578 V). C) Each electrode (n = 5) has its own sensitivity to DA, but all show a linear response to increasing concentrations of DA (Bare electrode *r* ^2^ range = 0.96102–0.99747, mean = 0.988758; solid lines). Upon exposure to wax/xylenes, all electrode best fit lines have increased in height (Post-wax/xylenes *r* ^2^ range = 0.97886–0.99815, mean = 0.992828; dashed lines). D) To better visualize this change in sensitivity and to account for differences in electrode sensitivity, all electrode responses to DA were normalized to the intra-electrode bare response to 2000 nM. The bare carbon fiber electrodes show a linear response to increasing concentrations of DA as do the electrodes after exposure to wax and then xylenes (*r* ^2^ = 0.99968 and 0.99897 for bare and post-wax/xylenes, respectively). The adjusted means (height of lines) differ (*p* = 0.003198), as do the slopes (*p* = 0.0001127), which would indicate an increase in sensitivity of the electrodes to DA due to wax/xylenes treatment.

To determine if exposure to paraffin wax dampens the electrode sensitivity to catecholamines, glass-sealed electrodes were calibrated with successive increases in [DA] (in aCSF). They were then dipped in molten paraffin and subsequently swirled in xylenes. Fig 4A shows that exposure to paraffin wax (prior to dipping in xylenes) reduces the BC, indicating a loss in reactive surface area. This same electrode was then dipped in xylenes, and cycled in aCSF; the BC has returned to the original BC size. Fig 4B demonstrates that exposure to wax and then xylenes does not foul the reaction of DA at the carbon surface, as the oxidation peak for DA (1000 nM, normalized for comparison) shifts only slightly (0.024 V) to the right.

Fig 4C shows that although each electrode of the five tested has a different nA/*μ*M response to DA (each color represents a different electrode; n = 5), they all show an increased sensitivity to DA after exposure to wax and then xylenes. Solid lines are fit to bare electrode calibrations, dashed lines are fit to post-wax/xylenes calibration data (Bare electrode *r* ^2^ range = 0.96102–0.99747, mean = 0.988758; Post-wax/xylenes *r* ^2^ range = 0.97886–0.99815, mean = 0.992828). When normalized to each electrodes’ peak bare electrode 2000 nM response (Fig 4D), it can be seen that there is a strong linearity in response to increasing concentrations of DA both before (bare *r* ^2^ = 0.99968) and after (post-wax/xylenes *r* ^2^ = 0.99897) exposure to wax and then xylenes. However, there was an increased overall sensitivity to DA after wax/xylenes exposure shown by an increase in line height and slope. A one-way ANCOVA determined that the adjusted means (line heights) differ significantly (*p* = 0.003198), and there is a significantly higher slope post-wax/xylenes (*p* = 0.0001127; bare slope = 0.40538, post-wax/xylenes slope = 0.63748). This demonstrates that not only does the electrode sensitivity not decrease after wax and xylenes exposure, it increases.

### Glass Encasement vs. Fused-Silica Encasement

**Fig 5:**
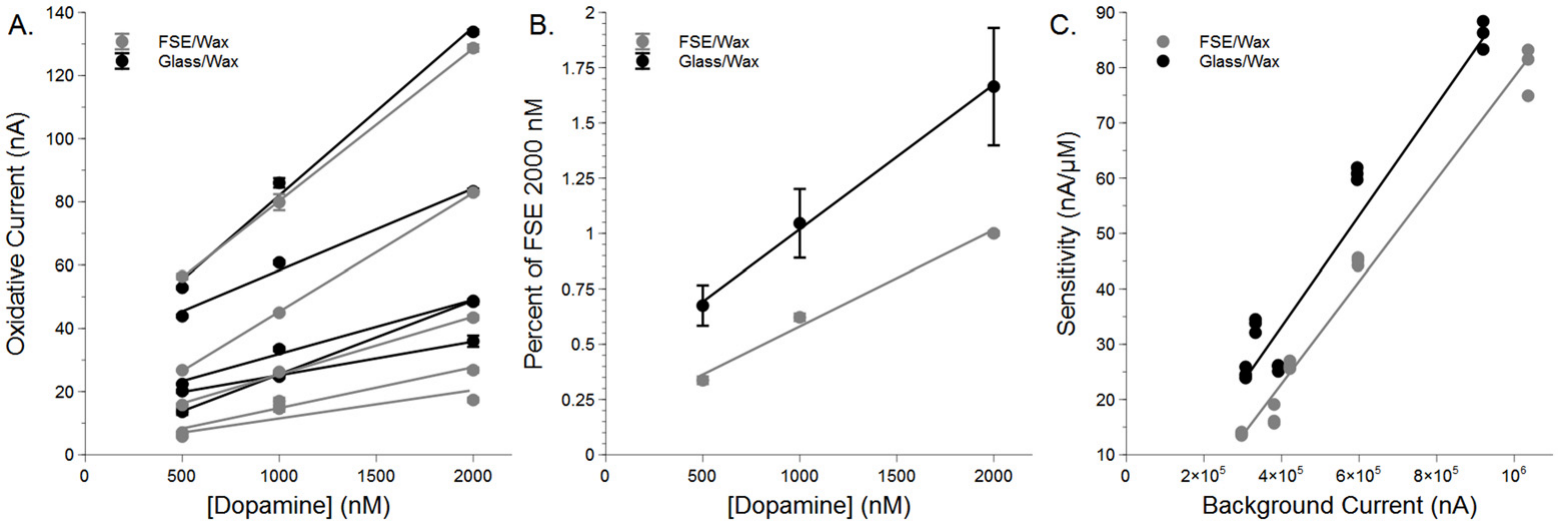
Encasement material affects DA sensitivity. Glass/wax encased electrodes are more sensitive to dopamine than fused-silica/wax electrodes. A) Peak currents in response to increasing concentrations of dopamine vary per electrode, but all electrodes respond in a linear fashion (*r* ^2^ range = 0.98833–0.99979, mean = 0.993608). B) All electrode responses to DA are normalized to the FSE/Wax peak at 2000 nM in order to compare Glass/Wax to FSE/Wax. For both conditions, a linear response to increasing [DA] (*r* ^2^ = 0.98789 and 0.99785 for FSE/Wax and Glass/Wax, respectively) can be seen. From the fit lines, one can see that Glass/Wax electrodes have a higher response to DA (p = 0.012; slopes do not differ, p = 0.0601). C) To incorporate the size of the electrode (indicated by background charging current, BC) into the discussion of sensitivity, the sum of all BC absolute values was plotted vs. sensitivity to DA (nA/*μ*M). Both encasement types produced a linear model indicating that sensitivity to DA is tied to total BC (*r* ^2^ = 0.98213 and 0.94892 for FSE and Glass, respectively). However, even though these lines do not differ in their slope (p = 0.1789), they do differ in their adjusted mean height (p < 0.0001), indicating that Glass/Wax electrodes have an increased sensitivity to DA over FSE/Wax.

Since both glass and fused-silica electrodes were used in this study, it is necessary to compare the impact of the encasement material (borosilicate glass or fused-silica) on DA sensitivity. Five electrodes from each group were exposed to increasing concentrations of [DA] (500, 1000, and 2000 nM). Similar to Fig 4C, each of the ten electrodes tested had varying sensitivities to [DA], but all showed a linear response to increasing [DA] (*r* ^2^ range = 0.98833–0.99979, mean = 0.993608). This can be seen in Fig 5A. When each electrode is normalized to the mean peak response at FSE 2000 nM, a linear mean response to DA becomes apparent (Fig 5B; FSE *r* ^2^ = 0.98789, Glass *r* ^2^ = 0.99785). What also emerges from this normalization is that the Glass-encased electrodes appear to demonstrate an increased sensitivity to DA (adjusted means differ p = 0.012, but slopes do not p = 0.0601). The large SEM seen in the Glass/Wax data is due to the variability between the Glass/Wax raw values and the FSE/Wax mean 2000 nM response, a tendency which can be seen in Fig 5A. When normalized to its own mean peak response at 2000 nM, the Glass/Wax SEM values are much smaller (data not shown).

Though normalizing to the 2000 nM response of the FSE electrodes indicates a difference in sensitivity, size of electrode and BC have a strong impact on sensitivity to DA [24]. To directly compare sensitivities of the two types of encasement, the sum of all the absolute values of BC were plotted vs. sensitivity (nA/*μ*M [24]). The result is seen in Fig 5C (n = 5 per group; 3 values per electrode). The slopes of the best fit lines (*r* ^2^ = 0.98213 and 0.94892 for FSE and Glass, respectively) do not differ from one another (p = 0.1789), but the height of the lines do (adjusted means differ p < 0.0001). This would indicate that Glass/Wax electrodes are more sensitive to DA than FSE/Wax electrodes.

### *In Vivo* use of Paraffin Sealant

**Fig 6:**
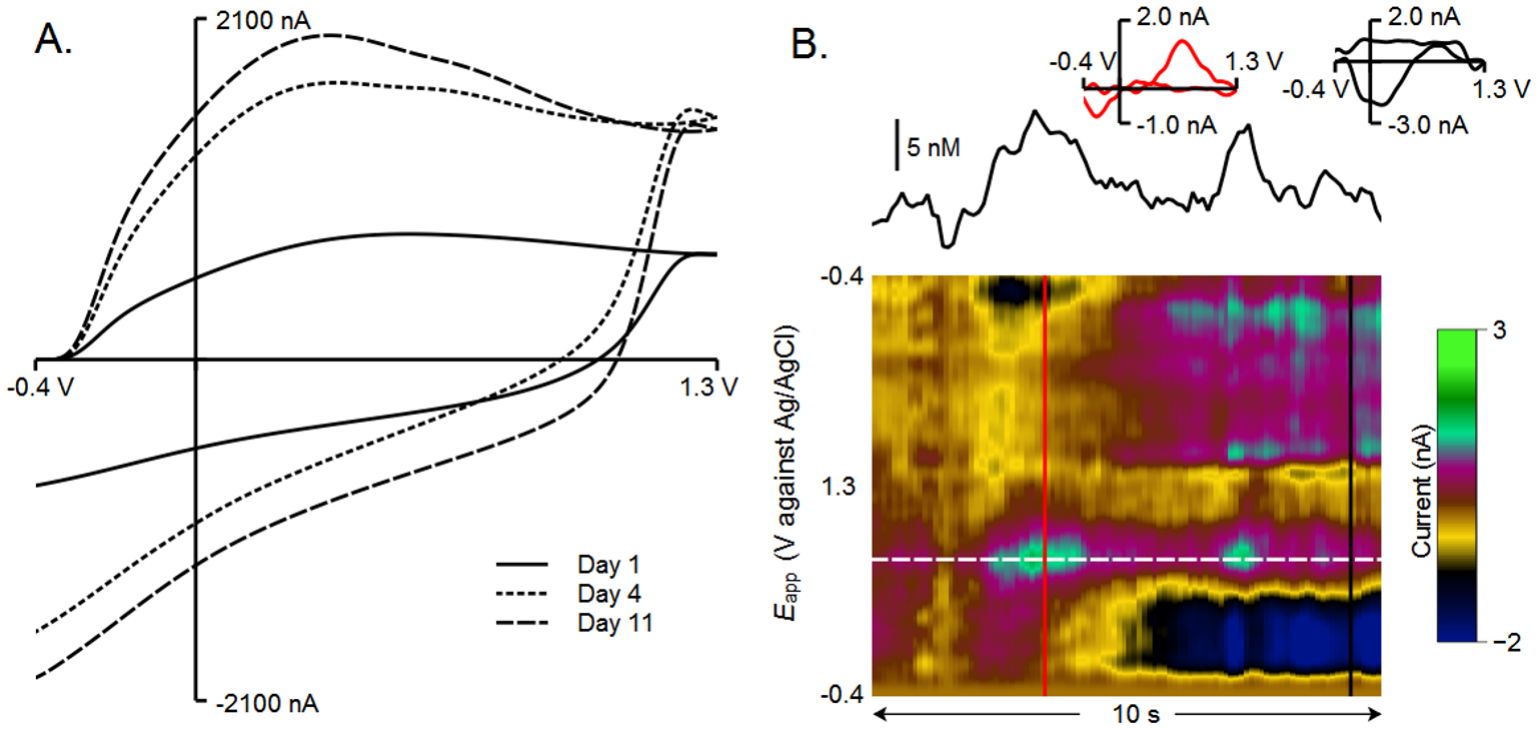
*In vivo* demonstration of wax-sealed electrodes. A fused-silica electrode (FSE) was sealed with embedding paraffin and implanted in the dorsal striatum of a female mouse. A) The background current of the electrode was still present at 4 and 11 days after implantation, after cycling from -0.4 to 1.3 V and back at 400 V/s and 60 Hz. B) Spontaneous dopamine transients were recorded, and can be seen in a pseudocolor plot of serial background-subtracted cyclic voltammograms. Above the pseudocolor is the current recorded at peak oxidation for dopamine converted to [DA], shown as a white dashed line in the color plot. A cyclic voltammogram was taken at the red line and is shown inset to the current recording, identifying the analyte as dopamine. Following this event, a pH change common to freely-moving preparations is detected and confirmed by the second inset.

Paraffin wax stability at mammalian body temperatures was assessed by sealing carbon fiber within fused-silica tubing and implanting it for 11 days in a female mouse. Fig 6A shows that upon repeated cycling (10 minutes, 60Hz, day 4; 45 minutes, 60Hz, day 11), the BC shape greatly resembles that seen in the previous figures, indicating an intact fused-silica/wax/carbon seal. Furthermore, this electrode measured naturally-occurring physiological changes in [DA] in real-time. The results are shown in Fig 6B in background-subtracted pseudocolor [25] (bottom). To verify that this signal is due to DA, a cyclic voltammogram taken at the red line shows the characteristic shape of DA oxidation and reduction (shown as the red inset). Also, a basic change in physiological pH can be seen at the location of the black line and is indicated by the black inset voltammogram.

Along the oxidation peak for DA (white dashed line) there are momentary increases in [DA], further simplified directly above the colorplot, where the black line shows only the changes in current along this oxidation peak. The calibration factor for this line was created by fitting the FSE data from Fig 5C to the equation described for *in situ* calibration [24]. The resulting fit was a good match between predicted and expected calibration data (data not shown; *r* ^2^ = 0.98213). The large peak seen in the beginning of this trace, based upon *in situ* calibration, would equate to ∼14.8 nM.

## Discussion

### Fabrication of Wax-Sealed Electrodes and Seal Stability

The almost immediate curing time of wax is a major advantage over epoxy resins and allows electrodes to be fabricated and tested straightaway. Glass-sealed electrodes have long-since boasted immediate testing times, however wax-sealed electrodes allow for two advantages. The first advantage is that wax-sealed electrodes provide a blatant end-of-seal, start-of-carbon distinction point, which allows for easier cutting of the carbon fiber to appropriate lengths. With glass-sealed electrodes, it can, at times, be difficult to determine the exact location of the glass/carbon seal [18]. The second advantage of wax-sealed over glass-sealed electrodes is that formerly unusable electrodes with a poor glass/carbon seal can be sealed with wax, which should increase the throughput of electrode creation, since electrodes normally thrown out can be salvaged. Creation of FSE, which heretofore required epoxy sealing, is also eased since surface fouling due to wax can be removed, whereas epoxy resin cannot (once hardened).

Even though sealing glass electrodes with wax is simple in its procedure, the glass seal must first be broken. This is the most difficult portion of creating wax-sealed electrodes, and appears to be very personalized, with each author preferring different methods. These include: cutting with a scalpel, breaking with the blunt end of a scalpel, cutting with scissors, or crushing with another capillary tube. In order to give more places for proper breaking due to its inherent variability, electrodes were pulled at normal heat with low pull strength, which resulted in long-tapered glass/carbon seals. This allowed many possible, and subsequent, spots to break the glass seal should early spots prove unfruitful. Through trial and error it was determined that jagged glass breaks led to angled wax seals, and resulted in fewer stable electrodes; straight-edge breaks appear to lead to more frequent stable electrodes. Also noted through trial and error is that smaller diameter wax seals seemed to yield a higher number of stable electrodes, albeit anectdotally. While the glass in Figs 1B or 2A are not perfectly perpendicular to the electrode’s longitudinal axis, they are not jagged, and are small diameter; these electrodes proved stable (data not shown; Fig 2B). Stability was determined by cycling the electrode with a ramp voltage of -0.4 to 1.3 to -0.4 V, 400 V/s, 60 Hz with data collection every 5 minutes, similar to Fig 2B. A stable electrode is seen as one whose BC discontinues rapid growth, and whose drift is small (< 2 nA per 30s).

It was common for the background charging current (BC), which results from the charging of the electrode double-layer [26] and changes at electrode surface groups [12, 27], to increase over time during initial cycling, possibly due to a thin layer of wax being cycled off of the electrode as the carbon surface is renewed, as demonstrated for other electrode-fouling materials by Takmakov and colleagues [5]. This would mean that as wax is cycled off the electrode, more surface reactions and a larger active surface area would result, leading to a larger BC. Eventually, the BC for stable electrodes discontinued this growth (similar to Fig 2B) whereas unstable electrodes had a continued increase in BC and additional random noise spikes developed, becoming more frequent over time (data not shown). Though we posit that the BC increases are indicative of a loss of wax off of the carbon surface, this may also be the result of the carbon surface activating with repeated cycling [28]. To expedite this process, electrodes can be dipped in xylenes to remove most (if not all) of the surface wax prior to cycling. In fact, the authors recommend using xylenes, followed by cycling, as it greatly speeds up the process (typically 15 minutes of cycling at 60 Hz is sufficient after xylenes; personal observation). Despite the fact that breaking and resealing sounds troublesome, each person seems to develop a rhythm and many individuals new to electrode creation develop stable electrodes in approximately two weeks or less, devoting roughly 2 hours per day (personal observation).

Since wax may be cycling off the carbon surface, it stands that there may be a risk of erosion of the glass/wax/carbon seal with repeated cycling. To demonstrate the stability of the seal for longer experimental recording times, the -0.4 to 1.3 V (and back at 400 V/s) waveform was chosen due to its frequent use *in vivo* [9, 10, 14, 20, 23], *ex vivo* [20], and *in vitro* [19, 29]. Typically, recordings are performed at 10 Hz [9, 10, 14, 20, 23, 29], but 60 Hz was chosen since its more frequent application would show any seal erosion 6*x* faster. Four hours of cycling, which would equate to 24 hours of 10 Hz cycling, proved that the wax seal remained stable. It is possible that the internal nature of the wax seal within the remaining glass insulates the loss of that particular wax from the carbon surface, or that the minimal carbon surface exposure to the surrounding milieu at the seal itself limits the rate of seal loss. In addition, the sheer size of the wax seal (∼ 6 mm in Fig 1B) should warrant extended protection from degradation.

The BC increased within this time, though this change is only a ∼7% increase over the equivalent of 24 cycling hours. A change in BC over time is not uncommon [30, 31], so a change in BC was anticipated. Due to the fact that this waveform application (60 Hz) will result in the full loss of the electrode within 65 hours [5], 4 hours is an order of magnitude lower than the time necessary to fully eliminate the carbon surface. 4 hours at 60 Hz or 24 hours at 10 Hz should be well within the realm of carbon fiber stability. This BC change, if troublesome, can be dealt with through either analog background subtraction [30] or principle component regression (PCR) [31]. At this time, the authors do not know of any study where 24 hours of 10 Hz cycling occurred, at least explicitly or continuously. It is possible, however, to achieve such lengthy cycling paradigms sequentially with multiple recordings at chronically-implanted microsensors [14]. Therefore, the wax seal is sufficient for most FSCV experimental protocols.

### Wax Sealing Does Not Interfere with Sensitivity

Exposure of the carbon fiber surface to a fouling substance will lead to dramatically reduced sensitivities to catecholamines [5, 29, 32]. To demonstrate that removal of surface wax with xylenes is sufficient to maintain sensitivity, glass-sealed electrodes were exposed an increasing concentration of DA through a gravity-fed calibration system [19]. The same electrodes were dipped in molten paraffin, it was allowed to cool, and then they were dipped in xylenes. The change in BC seen in Fig 4A after wax exposure shows that the surface is at least partially fouled by the paraffin wax. Even though almost full electrode fouling is possible (see Fig 3A), that is rare. Swirling the electrodes in xylenes, even after the wax has solidified, was sufficient to return the electrode to its full BC size. This change in size would not be due to cycling of the electrode, as minimal cycling was allowed post-wax (pre-xylenes) exposure. What is apparent is a slight change in the BC shape after xylenes exposure. This is likely due either to cycling or exposure to a solvent, as exposure to other solvents has been shown to clean the carbon surface [33].

Removing the wax with xylenes retained the carbon fiber’s sensitivity to DA, as the post-wax/xylenes electrodes were at least as sensitive to increasing concentrations of DA (Fig 3D). Both before and after wax/xylenes exposure, the electrodes showed a linear response to increasing [DA] up to 2000 nM, which is consistent with previous work [33–37]. To account for differences in electrode sensitivity, data were normalized to peak current response at 2000 nM for bare electrodes [19, 34]. The calibration after xylenes indicates that sensitivity to DA has increased after wax and xylenes exposure. The exact reason for this is unclear, but could potentially be the result of exposure to a solvent, which may clean the carbon surface and enhance sensitivity [33]. Regardless, this means that any surface wax remaining after molten wax exposure is removed from the active carbon surface by swirling the electrode in xylenes, thus sealing electrodes with wax does not interfere with their ability to detect catecholamines.

### Encasement Material Affects Sensitivity

As both glass and polyimide-encased fused-silica electrodes are commonly in use [9, 10, 14, 20, 23, 29, 38, 39], and a benefit of paraffin is its solidity at mammalian body temperatures, it stands that a direct comparison of Glass/Wax and FSE/Wax electrodes is warranted. Encasement materials have previously been shown to alter responses to DA, as Glass/Epoxy, FSE/Epoxy, or Epoxy alone electrodes each show differing peak oxidation voltage responses to the same concentration of DA [40]. Additionally, differing epoxies also showed slightly differing responses to DA [40]. Based upon this information, it was expected that FSE/Wax would be slightly more sensitive to DA. However, in our hands, Glass/Wax electrodes show higher sensitivity to DA, when monitored both by normalized calibrations curves (Fig 5B) and by direct sensitivity comparison (Fig 5C). While the reason for this difference is not obvious, it is possible that if Zestos and colleagues had accounted for differences in total BC [24], results similar to ours may have occurred. It is also possible that Glass/Epoxy, FSE/Epoxy, and Glass/Wax or FSE/Wax differ based upon sealants as to which encasement material yields a higher sensitivity.

Although the difference in sensitivity due to encasement material would appear important, in normal practice it may be of little issue. For instance, even though data here and elsewhere [40] would indicate potential differences in DA sensitivity due to encasement material, Glass-sealed and FSE/Epoxy electrodes demonstrate similar amplitudes for reward or cue-based DA events in freely-moving rats [14, 41]. In these instances, the accuracy of these values is more dependent upon proper calibration of the electrodes used. In a similar fashion, naturally occurring DA transients recorded in the mouse dorsal striatum in this study (discussed further below) are on par with those witnessed upon food delivery [42] even though the sealant and encasement materials differ (here: FSE/Wax, Natori *et al*.: Glass/Epoxy).

### Wax Seal *In vivo*

To demonstrate the stability of paraffin wax *in vivo*, a FSE was sealed with paraffin wax and implanted in a female mouse. The BC was monitored daily, and on days 4 and 11, the electrode was cycled to 1.3 V at 60 Hz to clean the carbon surface [5]. Fig 6A shows that after this cycling, the electrode is still intact as demonstrated by the intact BC. Should the electrode have lost its sealant, a much different BC shape would have resulted. This result also exhibits that the 50–53°C melting point of embedding paraffin is sufficient to withstand extended exposure to mammalian body temperature.

Within the 5 minute collection period following the extended cycling on day 11, transient DA events can be witnessed. Representative transients are shown in Fig 4B, and identified by the voltammogram for DA [43] (shown in red; upper right). This is also visible in serial voltammograms shown in pseudo-color [44] (bottom). Following the first DA event, a background shift consistent with a change in pH (second inset) is detectable. Large DA events are often followed by changes in pH and chemometric analysis typically includes a pH component, which is usually subtracted from recordings [45]. The ability of a wax-sealed FSE to record small, transient DA events further supports paraffin use as a FSCV sealant and shows that paraffin wax is a suitable sealant for use during *in vivo*, freely-moving FSCV applications. Embedding paraffin has additionally long been shown biocompatible in rodents [46].

### Conclusion

Herein we describe paraffin wax as a reliable sealant in the creation of glass and chronically implantable FSCV cylinder electrodes. Although “sticky paraffin” has been used previously in the creation of carbon fiber disc electrodes [47] this is the first time, to the authors’ knowledge, that it has been demonstrated and (more importantly) described for FSCV cylinder electrodes. Paraffin boasts several advantages over epoxy resin sealants. First, the paraffin can be melted again, which allows both the ability to reseal an electrode and the capability of reusing the same paraffin to seal numerous electrodes. Second, paraffin has an immediate curing time, which means electrodes can be tested immediately, which should decrease electrode output time. Third, paraffin-fouling of electrodes can be removed by cycling off of the carbon surface, or removing with xylenes, which both decrease the chances of electrode fouling. Lastly, not only is the paraffin seal stable after repeated cycling at the carbon surface, but it also remains stable at mammalian body temperatures over many days. Usage of wax to seal chronically implanted FSCV electrodes may be useful for future applications in studying the role of DA in learning, movement, and other cognitive functions.

## References

[1] Venton BJ, Zhang H, Garris PA, Phillips PE, Sulzer D, Wightman RM. Real-time decoding of dopamine concentration changes in the caudate-putamen during tonic and phasic firing. Journal of Neurochemistry. 2003;87:1284–1295. 14622108 10.1046/j.1471-4159.2003.02109.x

[2] Venton BJ, Wightman RM. Psychoanalytical electrochemistry: dopamine and behavior. Analytical Chemistry. 2003;75:414A–421A. 10.1021/ac031421c

[3] Park J, Takmakov P, Wightman RM. In vivo comparison of norepinephrine and dopamine release in rat brain by simultaneous measurements with fast-scan cyclic voltammetry. Journal of Neurochemistry. 2011;119:932–944. 10.1111/j.1471-4159.2011.07494.x 21933188 PMC3217157

[4] Hashemi P, Dankoski EC, Wood KM, Ambrose RE, Wightman RM. In vivo electrochemical evidence for simultaneous 5-HT and histamine release in the rat substantia nigra pars reticulata following medial forebrain bundle stimulation. Journal of Neurochemistry. 2011;118:749–759. 10.1111/j.1471-4159.2011.07352.x 21682723 PMC3155665

[5] Takmakov P, Zachek MK, Keithley RB, Walsh PL, Donley C, McCarty GS, et al Carbon microelectrodes with a renewable surface. Analytical Chemistry. 2010;82:2020–2028. 10.1021/ac902753x 20146453 PMC2838506

[6] Keithley RB, Takmakov P, Bucher ES, Belle AM, Owesson-White CA, Park J, et al Higher Sensitivity Dopamine Measurements with Faster-Scan Cyclic Voltammetry. Analytical Chemistry. 2011;83:3563–3571. 10.1021/ac200143v 21473572 PMC3089759

[7] Moquin KF, Michael AC. Tonic autoinhibition contributes to the heterogeneity of evoked dopamine release in the rat striatum. Journal of Neurochemistry. 2009;110:1491–1501. 10.1111/j.1471-4159.2009.06254.x 19627437 PMC2761222

[8] Ramsson ES, Covey DP, Daberkow DP, Litherland MT, Juliano SA, Garris PA. Amphetamine augments action potential-dependent dopaminergic signaling in the striatum in vivo. Journal of Neurochemistry. 2011;117:937–948. 10.1111/j.1471-4159.2011.07258.x 21443523 PMC3134290

[9] Ramsson ES, Howard CD, Covey DP, Garris PA. High doses of amphetamine augment, rather than disrupt, exocytotic dopamine release in the dorsal and ventral striatum of the anesthetized rat. Journal of Neurochemistry. 2011;119:1162–1172. 10.1111/j.1471-4159.2011.07407.x 21806614 PMC3213283

[10] Howard CD, Daberkow DP, Ramsson ES, Keefe KA, Garris PA. Methamphetamine-induced neurotoxicity disrupts naturally occurring phasic dopamine signaling. European Journal of Neuroscience. 2013;38:2078–2088. 10.1111/ejn.12209 23574406 PMC3699967

[11] Jacobs CB, Vickrey TL, Venton BJ. Functional groups modulate the sensitivity and electron transfer kinetics of neurochemicals at carbon nanotube modified microelectrodes. The Analyst. 2011;136:3557–3565. 10.1039/c0an00854k 21373669 PMC4169050

[12] Kawagoe KT, Zimmerman JB, Wightman RM. Principles of voltammetry and microelectrode surface states. Journal of Neuroscience Methods. 1993;48:225–240. 10.1016/0165-0270(93)90094-8 8412305

[13] Ponchon JL, Cespuglio R, Gonon F, Jouvet M, Pujol JF. Normal pulse polarography with carbon fiber electrodes for in vitro and in vivo determination of catecholamines. Analytical Chemistry. 1979;51:1483–1486. 10.1021/ac50045a030 484865

[14] Clark JJ, Sandberg SG, Wanat MJ, Gan JO, Horne EA, Hart AS, et al Chronic microsensors for longitudinal, subsecond dopamine detection in behaving animals. Nature Methods. 2010;7:126–129. 10.1038/nmeth.1412 20037591 PMC2849934

[15] Bergstrom BP, Schertz KE, Weirick T, Nafziger B, Takacs SA, Lopes KO, et al Partial, graded losses of dopamine terminals in the rat caudate-putamen: an animal model for the study of compensatory adaptation in preclinical parkinsonism. Journal of Neuroscience Methods. 2001;106:15–28. 10.1016/S0165-0270(00)00372-1 11248337

[16] Wightman RM. Microvoltammetric electrodes. Analytical Chemistry. 1981;53:1125A–1134A. 10.1021/ac00232a791 22742042

[17] Cahill PS, Walker QD, Finnegan JM, Mickelson GE, Travis ER, Wightman RM. Microelectrodes for the measurement of catecholamines in biological systems. Analytical Chemistry. 1996;68:3180–3186. 10.1021/ac960347d 8797378

[18] Fortin SM, Cone JJ, Ng-Evans S, McCutcheon JE, Roitman MF. Sampling Phasic Dopamine Signaling with Fast-Scan Cyclic Voltammetry in Awake, Behaving Rats In: Current Protocols in Neuroscience. John Wiley & Sons, Inc; 2015. 10.1002/0471142301.ns0725s70 PMC4311885 25559005

[19] Sinkala E, McCutcheon JE, Schuck MJ, Schmidt E, Roitman MF, Eddington DT. Electrode calibration with a microfluidic flow cell for fast-scan cyclic voltammetry. Lab on a Chip. 2012;12:2403–2408. 10.1039/c2lc40168a 22522908 PMC3371170

[20] Yorgason JT, España RA, Jones SR. Demon voltammetry and analysis software: analysis of cocaine-induced alterations in dopamine signaling using multiple kinetic measures. Journal of Neuroscience Methods. 2011;202:158–164. 10.1016/j.jneumeth.2011.03.001 21392532 PMC3149733

[21] Paxinos G, Franklin KBJ. The Mouse Brain in Stereotaxic Coordinates, Compact, Third Edition: The coronal plates and diagrams. 3rd ed Amsterdam: Academic Press; 2008.

[22] Jin X, Costa RM. Start/stop signals emerge in nigrostriatal circuits during sequence learning. Nature. 2010;466:457–462. 10.1038/nature09263 20651684 PMC3477867

[23] Cheer JF, Wassum KM, Sombers LA, Heien ML, Ariansen JL, Aragona BJ, et al Phasic dopamine release evoked by abused substances requires cannabinoid receptor activation. The Journal of Neuroscience. 2007;27:791–795. 10.1523/JNEUROSCI.4152-06.2007 17251418 PMC6672925

[24] Roberts James G. and Toups J. Vincent and Eyualem Eyob and McCarty Gregory S. and Sombers Leslie A. In Situ Electrode Calibration Strategy for Voltammetric Measurements In Vivo Analytical Chemistry. 2013;85:11568–11575. 10.1021/ac402884n 24224460 PMC3935327

[25] Michael DJ, Joseph JD, Kilpatrick MR, Travis ER, Wightman RM. Improving data acquisition for fast-scan cyclic voltammetry. Analytical Chemistry. 1999;71:3941–3947. 10.1021/ac990491+ 10500480

[26] Bard AJ, Faulkner LR. Electrochemical methods. Wiley, New York; 2001.

[27] Runnels PL, Joseph JD, Logman MJ, Wightman RM. Effect of pH and surface functionalities on the cyclic voltammetric responses of carbon-fiber microelectrodes. Analytical Chemistry. 1999;71:2782–2789. 10.1021/ac981279t 10424168

[28] Hsueh C, Bravo R, Jaramillo AJ, Brajter-Toth A. Surface and kinetic enhancement of selectivity and sensitivity in analysis with fast scan voltammetry at scan rates above 1000 V/s. Analytica Chimica Acta. 1997;349:67–76. 10.1016/S0003-2670(97)00209-2

[29] Cooper SE, Venton BJ. Fast-scan cyclic voltammetry for the detection of tyramine and octopamine. Analytical and Bioanalytical Chemistry. 2009;394:329–336. 10.1007/s00216-009-2616-0 19189084

[30] Hermans A, Keithley RB, Kita JM, Sombers LA, Wightman RM. Dopamine detection with fast-scan cyclic voltammetry used with analog background subtraction. Analytical Chemistry. 2008;80:4040–4048. 10.1021/ac800108j 18433146

[31] Keithley RB, Wightman RM. Assessing Principal Component Regression Prediction of Neurochemicals Detected with Fast-Scan Cyclic Voltammetry. ACS Chemical Neuroscience. 2011;2:514–525. 10.1021/cn200035u 21966586 PMC3182154

[32] Jackson BP, Dietz SM, Wightman RM. Fast-scan cyclic voltammetry of 5-hydroxytryptamine. Analytical Chemistry. 1995;67:1115–1120. 10.1021/ac00102a015 7717525

[33] Bath BD, Michael DJ, Trafton BJ, Joseph JD, Runnels PL, Wightman RM. Subsecond adsorption and desorption of dopamine at carbon-fiber microelectrodes. Analytical Chemistry. 2000;72:5994–6002. 10.1021/ac000849y 11140768

[34] Heien ML, Phillips PE, Stuber GD, Seipel AT, Wightman RM. Overoxidation of carbon-fiber microelectrodes enhances dopamine adsorption and increases sensitivity. The Analyst. 2003;128:1413–1419. 14737224 10.1039/b307024g

[35] Addy NA, Daberkow DP, Ford JN, Garris PA, Wightman RM. Sensitization of rapid dopamine signaling in the nucleus accumbens core and shell after repeated cocaine in rats. Journal of Neurophysiology. 2010;104:922–931. 10.1152/jn.00413.2010 20554845 PMC2934942

[36] Sterky FH, Hoffman AF, Milenkovic D, Bao B, Paganelli A, Edgar D, et al Altered dopamine metabolism and increased vulnerability to MPTP in mice with partial deficiency of mitochondrial complex I in dopamine neurons. Human Molecular Genetics. 2012;21:1078–1089. 10.1093/hmg/ddr537 22090423 PMC3277308

[37] Diaz-Ruiz O, Zhang Y, Shan L, Malik N, Hoffman AF, Ladenheim B, et al Attenuated response to methamphetamine sensitization and deficits in motor learning and memory after selective deletion of β-catenin in dopamine neurons. Learning & Memory. 2012;19:341–350. 10.1101/lm.026716.112 22822182 PMC3407939

[38] Bucher Elizabeth S. and Wightman R. Mark Electrochemical Analysis of Neurotransmitters Annual Review of Analytical Chemistry. 2015;8:239–261. 10.1146/annurev-anchem-071114-040426 PMC4728736 25939038

[39] Oleson Erik B. and Gentry Ronny N. and Chioma Vivian C. and Cheer Joseph F. Subsecond Dopamine Release in the Nucleus Accumbens Predicts Conditioned Punishment and Its Successful Avoidance The Journal of Neuroscience. 2012;32:14804–14808. 10.1523/JNEUROSCI.3087-12.2012 23077064 PMC3498047

[40] Zestos Alexander G. and Nguyen Michael D. and Poe Brian L. and Jacobs Christopher B. and Venton B. Jill Epoxy insulated carbon fiber and carbon nanotube fiber microelectrodes 2013;182:652–658. 10.1016/j.snb.2013.03.066 PMC8081386 33927480

[41] Daberkow D. P. and Brown H. D. and Bunner K. D. and Kraniotis S. A. and Doellman M. A. and Ragozzino M. E. and Garris P. A. and Roitman M. F. Amphetamine paradoxically augments exocytotic dopamine release and phasic dopamine signals The Journal of Neuroscience. 2013;33:452–463. 10.1523/JNEUROSCI.2136-12.2013 23303926 PMC3711765

[42] Natori Shihoko and Yoshimi Kenji and Takahashi Toshimitsu and Kagohashi Maki and Oyama Genko and Shimo Yasushi and Hattori Nobutaka and Kitazawa Shigeru Subsecond reward-related dopamine release in the mouse dorsal striatum Neuroscience research. 2009;63:267–272. 19367786 10.1016/j.neures.2008.12.011

[43] Baur JE, Kristensen EW, May LJ, Wiedemann DJ, Wightman RM. Fast-scan voltammetry of biogenic amines. Analytical Chemistry. 1988;60:1268–1272. 10.1021/ac00164a006 3213946

[44] Michael D, Travis ER, Wightman RM. Color images for fast-scan CV measurements in biological systems. Analytical Chemistry. 1998;70:586A–592A. 10.1021/ac9819640 9737201

[45] Keithley RB, Heien ML, Wightman RM. Multivariate concentration determination using principal component regression with residual analysis. Trends in Analytical Chemistry. 2009;28:1127–1136. 10.1016/j.trac.2009.07.002 20160977 PMC2760950

[46] Bryan GT, Brown RR, Price JM. Incidence of Mouse Bladder Tumors Following Implantation of Paraffin Pellets Containing Certain Tryptophan Metabolites. Cancer Research. 1964;24:582–585. 14188459

[47] Staal RGW, Rayport S, Sulzer D. Amperometric Detection of Dopamine Exocytosis from Synaptic Terminals In: Michael AC, Borland LM, editors. Electrochemical Methods for Neuroscience. Boca Raton (FL): CRC Press; 2007. 21204391

